# Plumbagin induces RPE cell cycle arrest and apoptosis via p38 MARK and PI3K/AKT/mTOR signaling pathways in PVR

**DOI:** 10.1186/s12906-018-2155-3

**Published:** 2018-03-13

**Authors:** Haiting Chen, Huifang Wang, Jianbin An, Qingli Shang, Jingxue Ma

**Affiliations:** 1 Department of Ophthalmology, Second Hospital of Hebei Medical University, No. 215 Peace West Road, Qiaoxi District, Shijiazhuang, Hebei 050000 China; 2 Department of Ophthalmology, Cangzhou Central Hospital, No. 16 Xinhua West Road, Cangzhou, Hebei 061000 China

**Keywords:** RPE, Plumbagin, Proliferation

## Abstract

**Background:**

This study aimed to explore the effects of plumbagin (PLB) on ARPE-19 cells and underlying mechanism.

**Methods:**

Cultured ARPE-19 cells were treated with various concentrations (0, 5, 15, and 25 μM) of PLB for 24 h or with 15 μM PLB for 12, 24 and 48 h. Then cell viability was evaluated by MTT assay and DAPI staining, while apoptosis and cell cycle progression of ARPE cells were assessed by flow cytometric analysis. Furthermore, the level of main regulatory proteins was examinated by Western boltting and the expression of relative mRNA was tested by Real-Time PCR.

**Results:**

PLB exhibited potent inducing effects on cell cycle arrest at G2/M phase and apoptosis of ARPE cells via the modulation of Bcl-2 family regulators in a concentration- and time-dependent manner. PLB induced inhibition of phosphatidylinositol 3-kinase (PI3K) and p38 mitogen-activated protein kinase (p38 MAPK) signaling pathways contributing to the anti-proliferative activities in ARPE cells.

**Conclusions:**

This is the first report to show that PLB could inhibit the proliferation of RPE cells through down-regulation of modulatory signaling pathways. The results open new avenues for the use of PLB in prevention and treatment of proliferative vitreoretinopathy.

## Background

Proliferative vitreoretinopathy (PVR) is characterized by proliferation of cells and contraction of membranes on either retinal surface or in the vitreous cavity, which leads to retinal detachment and visual impairment [1]. It remains the most significant obstacle to successful retinal reattachment surgery, with a cumulative risk of 5% to 10% of all retinal detachment repairs, leading to poor functional results [2]. The most important cell type in PVR pathogenesis is the retinal pigment epithelial (RPE) cell, which is deemed to dedifferentiate and migrate through a retinal break and then proliferate on the retinal layers and vitrea, resulting in formation of epiretinal membranes [3].

At present, vitrectomy is still the main method of PVR treatment. Although the equipment and operation skills have made tremendous progress in recent years, the surgical treatment is not ideal, and can not avoid recurrence, since vitrectomy itself is one of the common cause of PVR [4]. Therefore, more attempts have been made to develop nonsurgical therapies for PVR, especially pharmacological strategies.

Plumbagin (PLB; 5-hydroxy-2-methyl-1,4-naphthoquinone) is a kind of natural naphthoquinone component isolated from the root of *Plumbago zeylanica* L, which has a extensive range of effects including anti-inflammatory, anti-microbial, anti-cancer, anti-atherosclerotic, and neuroprotective in multiple cell lines and animal models [5]. Recently, the anti-proliferative effect of PLB has been a hot research topic. It has been proved in several studies that this effect may cause cell cycle arrest and apoptosis [6–9].

In the present study, we aimed to investigate whether PLB can effectively inhibit proliferation of human RPE (ARPE-19) cells in vitro and find out the underlying mechanism.

## Methods

### Cell culture and treatment

A human RPE cell line (ARPE-19) was purchase from American Type Culture Collection (ATCC; Manassas, VA, USA) and cultured in a DMEM/F12 medium supplemented with 10% FBS and regular antibiotics (1% penicillin and streptomycin) (Gibco®; Thermo Fisher Scientific, Inc., Waltham, MA, USA) at 37 °C in a humidified atmosphere with 5% CO_2_ with medium changed every 3 days. Early-passage cells (6-8th passage) were used in the following experiments. Plumbagin (PLB; Sigma-Aldrich) was dissolved in dimethyl sulfoxide (DMSO; Sigma-Aldrich) and stocked at 100 mM, which was diluted to working concentrations with culture medium. ARPE-19 cells were cultured under two conditions: (1) with various concentration of plumbagin (0, 5, 15 or 25 μM) for 24 h; or (2) with plumbagin at 15 μM for 12, 24 and 48 h. The control cells received the vehicle (0.05% DMSO) only.

### Microscopic studies

ARPE-19 cells with PLB in various concentration were seeded in culture dishes and observed under an inverted microscope (Axiovert 200, Zeiss; Oberkochen, Germany). Then cells were fixed in 4% paraformaldehyde solution, then stained with 10 μg/ml 4, 6-diamidino-2-phenolindole (DAPI; Sigma- Aldrich) to display the nuclei under a fluorescence microscope (BX53TR, Olympus; Japan).

### Cell viability and proliferation assay

The 3-(4, 5 dimethyl-thiazol-2-yl)-2, 5-diphenyltetrazolium bromide (MTT) assay was performed to assess the effect of PLB on the viability of ARPE-19. Briefly, the ARPE cells were trypsinized, centrifuged, and seeded in 96-well (Thermo Fisher Scientific, Inc.) at a density of 8 × 10^3^ cells/well. After PLB treatment, cells in each well were incubated with 20 μL of MTT (5 mg/mL) for a further 4 h, then the crystals were dissolved with 150 μL DMSO by shaking slowly for 10 min. The absorbance was determined at the wavelengths of 540 nm using a fluorescence spectrophotometer (RF-6000, shimadzu; Japan).

### Assessment of cellular apoptosis

The Annexin V-FITC/PI apoptosis detection kit (BD Biosciences Inc.; San Jose, CA, USA) were used to measure the number of apoptotic cells after ARPE cells were treated with PLB. Briefly, cells were trypsinized and collected at the indicated time points, then adjusted to concentration at 1 × 10^6^/ml, resuspended in 500 μl buffer containing 5 μl Annexin V-FITC, 5 μl PI and incubated for 15 min in the dark at room temperature. The apoptotic cells were analyzed by FACSCalibur Flow Cytometer (Becton, Dickinson and Company; CA, USA) within 1 h.

### Cell cycle distribution analysis

After treatment as described previously, the cells were harvested and fixed with cold 70% ethanol. Next, 100 μl RNase A (25 μg/mL) and 400 μl (50 μg/mL) PI (DNA stainer; Sigma Aldrich; St. Louis, MO, USA) were added and incubated for 30 min in the dark room. At last, 1 × 10^4^ cells of each sample were analyzed by a flow cytometer (Becton, Dickinson and Company; San Jose, CA, USA) at the wavelengths of 488 nm.

### Western blot and ELISA

The expression level of proliferative related proteins were assessed by Western blotting assays. The treated ARPE cells were lysed with RIPA buffer (Solario; Beijing, China) and protein contents were determined by Pierce™ bicinchoninic acid (BCA) protein assay kit (Thermo Fisher Scientific Inc.; MA, USA). Each protein sample at 50 ng and rainbow molecular weight markers (11–245 kDa, Solario, Beijing, China) were electrophoresed on 8%–10% sodium dodecyl sulfate polyacrylamide gel electrophoresis minigel (SDS-PAGE) after thermal denaturation at 95 °C for 5 min. Proteins were electrotransferred onto polyvinylidene difluoride (PVDF) membranes (EMD Millipore; Billerica, MA, USA) at 100 V at 4 °C for 2 h, then blocked with 5% non-fat milk. Subsequently, membranes were probed with indicated primary antibody against Bak (ab32371), Bax(ab32503), Bcl-2(ab32124), Bcl-x(ab32370), p38 (phospho T180; ab178867), p-PI3K (phospho Y607; ab182651), β-Catenin(ab6302) and Notch1(ab83232) (Abcam; Cambridge,UK) at 4 °C overnight and then blotted with goat anti-rabbit horseradish peroxidase-conjugated secondary antibody (IgG; catalog no. 7074S; Cell Signaling Technology Inc.; MA, USA) for 1 h at room temperature according to the manufacturer’s protocol. The protein bands were visualized using the Bio-Rad Chemi Doc™ XRS system (Bio-Rad Laboratories Inc.; CA, USA) and band density was measured using Image J image analytical software (National Institutes of Health,Bethesda, MD). The protein concentration was calculated based on the value of the internal control β-actin (ab32572) or glyceraldehyde-3-phosphate dehydrogenase (GAPDH; ab128915; Abcam; Cambridge, UK). Under the same cultured conditions, the level of Bax, Cytochrome C, Caspase-3 and Caspase-8 were determined by ELISA kits (Roche Biochemicals) following the manufacturer’s instructions.

### RNA extraction and real-time PCR

ARPE-19 cells were cultured and treated as described previously. Afterwards, total RNA from 6 × 10^5^ cells was extracted collected with Trizol reagent (Invitrogen; Carlsbad, USA) and quantified by spectrophotometry. Reverse transcription was then performed with RNA reverse transcription kit (TaKaRa; Japan). Afterwards, PCR was carried out with SYBR Green qRT-PCR kit (Thermo Fisher Scientific; MA, USA) according to the manufacturer’s instructions for 40 cycles. Each cycle was composed of 30 s at 95 °C, 20 s at 55 °C and 20 s at 72 °C. The expression level of β-actin was set as control, whereas the mRNA expression level ΔΔCt = PLB group [Ct (objective)-Ct (β-actin)]-control group [Ct (objective)-Ct (β-actin)].

### Statistical analysis

The experiments were performed at least three times and data were expressed as mean ± standard deviation (SD). SPSS version 22.0 (SPSS, Inc.; Chicago, USA) was used to perform statistical analyses. Differences between control and treated samples were evaluated by one-wayanalysis of variance (ANOVA) followed by Tukey’s multiple comparison procedure. And a Pearson test was performed for correlation. A value of *p* < 0.05 was considered as statistically significant in each experiment.

## Results

### PLB inhibits the viability of ARPE cells

**Fig. 1:**
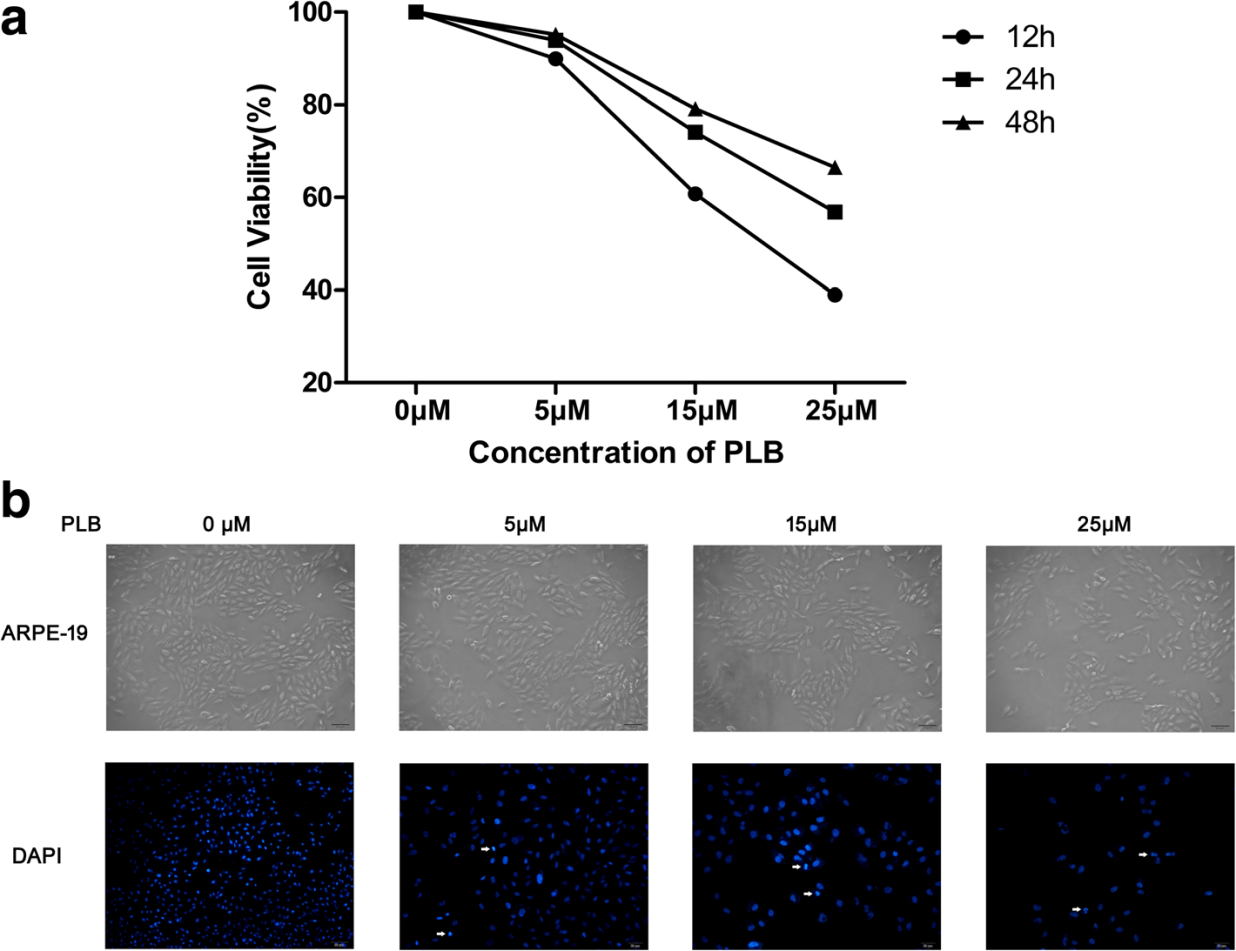
Effects of plumbagin on the viability of ARPE cells. Notes: (**a**) ARPE cells were treated with indicated concentrations of plumbagin for 24 h and cell survival was measured by MTT assay. The results are expressed as percentage of control; (**b**) The morphological characteristic of cells and nuclears were analyzed with phase-contrast image (Scale bar 50 μm) and DAPI staining (Scale bar 20 μm). Arrows indicated apoptotic morphological changes (e.g., chromatin condensation, shrinkage and nuclear fragmentation). Figures were selected as representative data from three independent experiments

The effect of PLB on viability of ARPE cells was evaluated by MTT assay. In comparison to the control cells, the viability of cells exposed to PLB at 5, 15 or 25 μM for 12 h were 95.17%, 79.18% and 66.53%; 93.92%, 74.11% and 56.81% at 24 h; 89.88%, 60.77% and 38.94% at 48 h, respectively (Fig. 1a). The IC50 were 30.94, 26.67 and 20.43 μM for ARPE cells after 12, 24 and 48 h’ incubation with PLB, respectively.

In parallel, treated with various concentration of PLB after 24 h, the ARPE cells appeared typical apoptosis morphological changes, such as cell shrinkage, lost normal spindle shape or broken under inverted optical microscope. The number of cells decreased with the increase of drug concentration. Furthermore, the nucleus pycnosis and cracking could be observed after DAPI staining, especially in cells with high concentration of PLB (Fig. 1b).

To sum up, these results proved that PLB had a potent anti-proliferative effect on ARPE cells.

### PLB induces apoptosis of ARPE cells

**Fig. 2:**
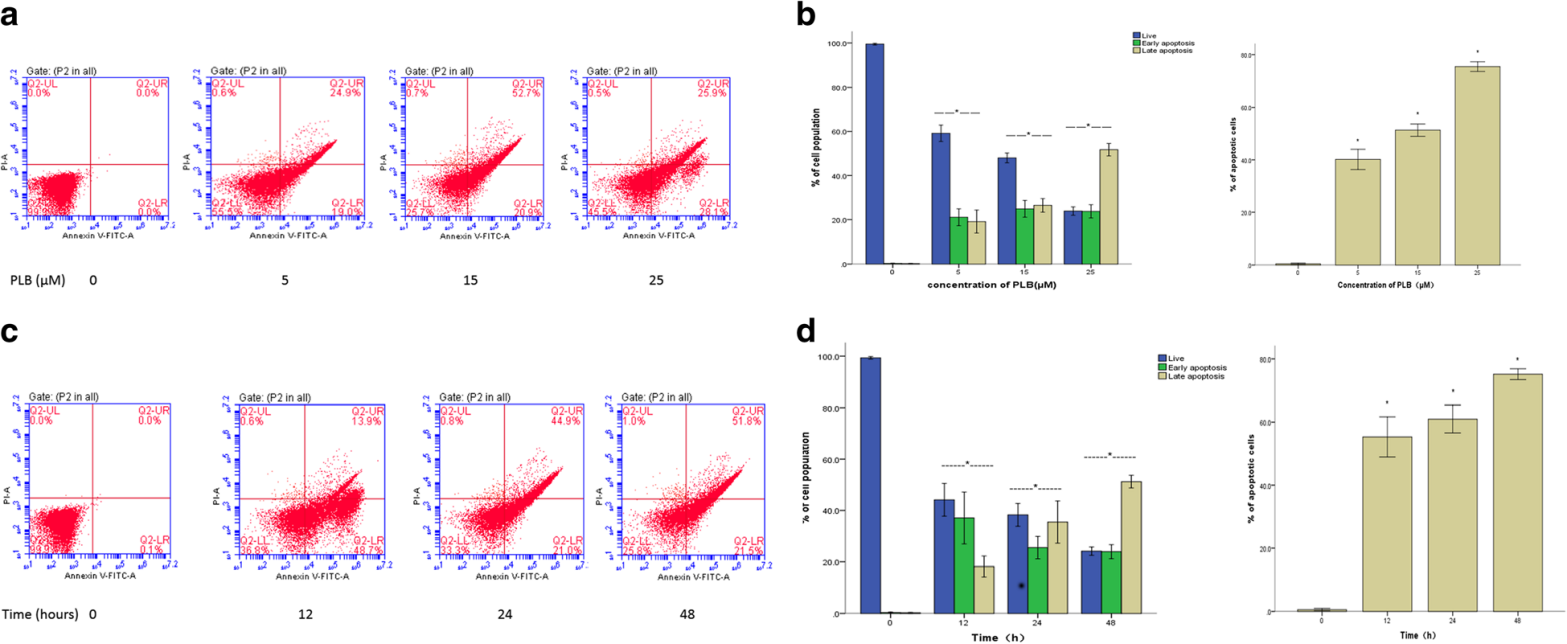
Plumbagin enhanced apoptosis of ARPE cells. The apoptosis of cells was analyzed by Annexin V-FITC/PI assay. Data were represented the mean ± SD of three individual experiments. (*P < 0.05 by one-way ANOVA.). Notes: (**a**) Flow cytometric plots show cells in the live, early apoptosis, and late apoptosis stages when the cells were treated with PLB at 0, 5, 15 and 25 μM for 24 h; (**b**) Bar graphs show the percentage of specific cell populations (live, early apoptosis, or late apoptosis) and total apoptosis cell population when the cells were treated with PLB at 0, 5, 15, and 25 μM for 24 h; (**c**) Flow cytometric plots show cells in the live, early apoptosis, and late apoptosis stages when the cells were treated with 15 μM PLB at 0, 12, 24,and 48 h; (**d**) Bar graphs show the percentage of specific cell populations (live, early apoptosis, or late apoptosis) and total apoptosis cell population when the cells were treated with 15 μM PLB at 0, 12, 24, and 48 h

The typical morphological changes occurred during the late apoptosis in ARPE cells have been demonstrated, so next we measured the effect of PLB on early apoptosis using Annexin V-FITC/PI staining analysis. After treated with PLB at 5, 15, and 25 μM for 24 h, the total percentage of apoptotic cells including early and late apoptosis stages were (40.16 ± 3.86)%, (51.33 ± 2.36)% and (75.43 ± 1.85)%, respectively. (Fig. 2a). Particularly, there was a remarkable rise in early, late or total apoptotic numbers when cells were incubated with 5 μM PLB or more (*P* < 0.001; Fig. 2b), and this progress was concentration-dependent (Pearson test; *P*<0.001). Furthermore, when ARPE cells were treated with 15 μM PLB for 12, 24, and 48 h (Fig. 2c), the percentage of apoptotic cells was increased from (0.57 ± 0.40)% at the basal line (time 0) to (55.30 ± 6.37)%, (60.97 ± 4.39)% and (75.13 ± 1.72)%, time-dependently (*P*<0.001, Fig. 2d; Pearson test, *P* = 0.001). These results indicated that PLB could induce apoptosis of ARPE cells prominently in a concentration- and time-dependent manner.

### PLB causes G2/M arrest in ARPE cells

**Fig. 3:**
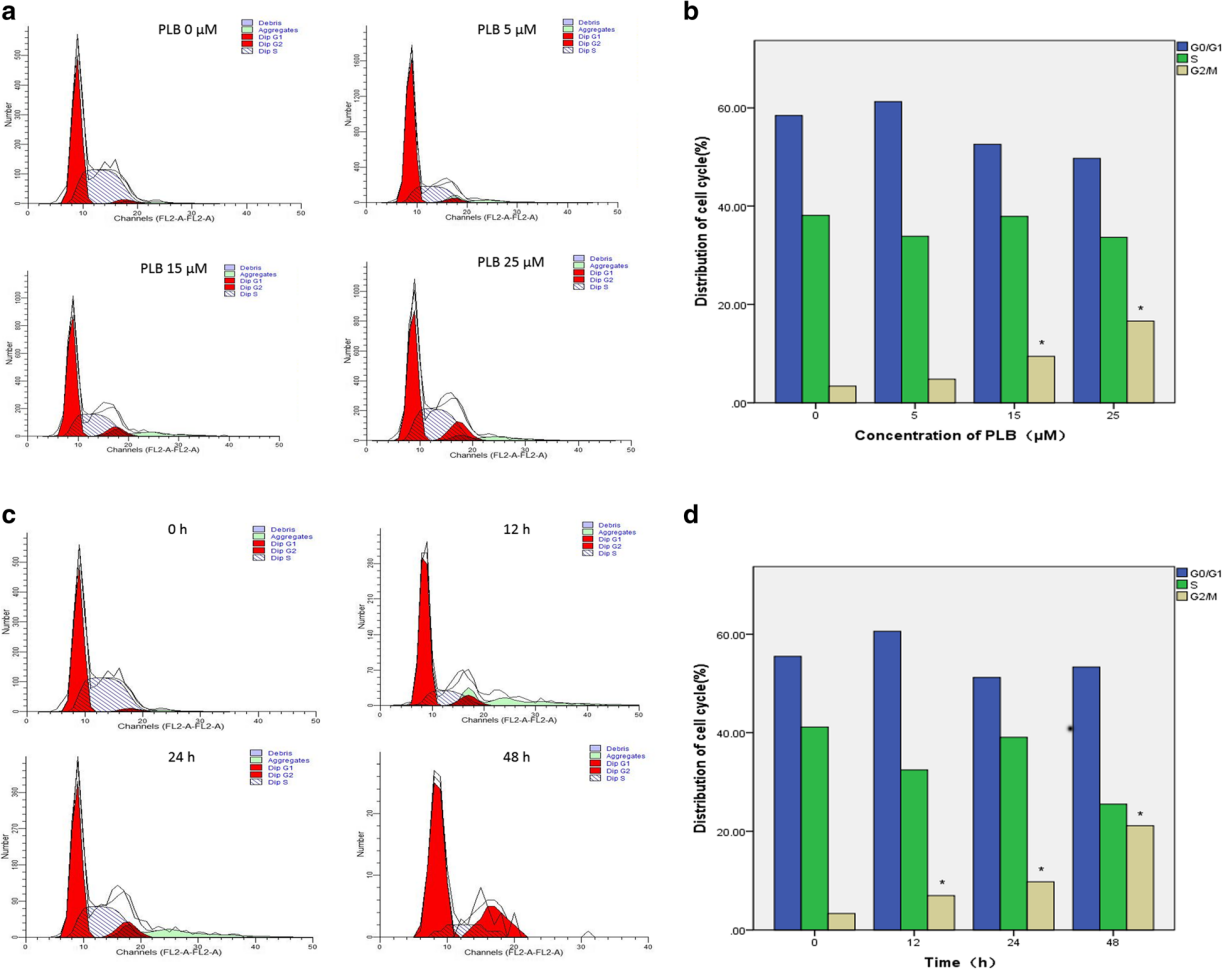
Plumbagin induces G2/M in ARPE cells. The population of cells was analyzed by flow cytometry. Data represented the mean ± SD of three individual experiments. (* *P* < 0.05 by one-way ANOVA.). Notes: (**a**) Flow cytometric histograms show the cell cycle distribution when the cells were treated with PLB at 0, 5, 15 and 25 μM for 24 h; (**b**) Bar graphs show the cell cycle distribution (G0/G1、S and G2/M phase) when the cells were treated with PLB at 0, 5, 15, and 25 μM for 24 h. **c** Flow cytometric histograms show the cell cycle distribution when the cells were treated with 15 μM PLB at 0, 12, 24, and 48 h; (**d**) Bar graphs show the cell cycle distribution (G0/G1、S and G2/M phase) when the cells were treated with 15 μM PLB at 0, 12, 24, and 48 h

In order to further explore the anti-proliferative mechanism of PLB on ARPE cells, we examined the cell cycle distribution using flow cytometric analysis. Cell cycle distribution showed no significant change at 5 μM for 24 h (*P* = 0.850), whereas the percentage of G2/M phase increased significantly in 15 and 25 μM groups (*P* = 0.038 and 0.000; Fig. 3a+b). Meanwhile, there was a 1.4-, 2.8-, and 4.9- fold increase in number of G2/M phase compared to the control group after cells were incubated with 15 μM PLB for 12, 24, and 48 h, respectively (*P* = 0.026,0.001,0.000; Fig. 3c+d). Furthermore, the results showed this progress was concentration-dependent (Pearson test; *P*<0.001) and time-dependent (Pearson test; *P* = 0.001). However, PLB treatments did not significantly affect the G0/G1 and S phases at the same time.

### PLB modulates key apoptotic regulators in ARPE cells

**Fig. 4:**
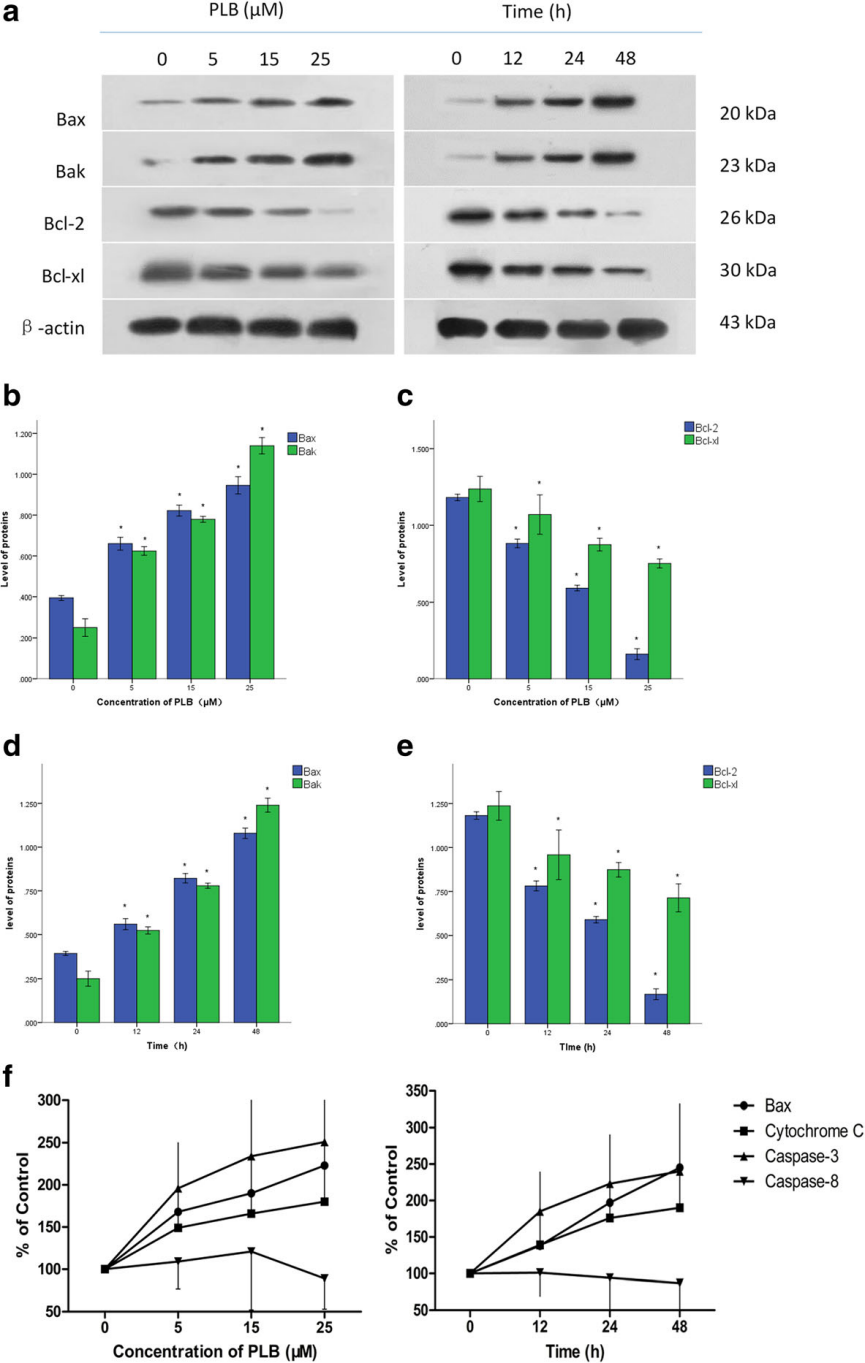
Effect of plumbagin on the expression of apoptosis-related proteins in ARPE cells. (*:*P* < 0.05 by one-way ANOVA,*n* = 3). Notes: (**a**) The expression levels of Bax, Bak, Bcl-2 and Bcl-xl determined by Western blotting assay when ARPE cells were treated with plumbagin at 0,5,15 and 25 μM. β-actin was used as the internal control. **b** Bar graphs show the expression levels of Bax and Bak when ARPE cells were treated with plumbagin at 0,5,15 and 25 μM for 24 h. **c** Bar graphs show the expression levels of Bcl-2 and Bcl-xl when ARPE cells were treated with plumbagin at 0,5,15 and 25 μM for 24 h. **d** Bar graphs show the expression levels of Bax and Bak when ARPE cells were treated with plumbagin at 15 μM for 0, 12, 24 and 48 h. **e** Bar graphs show the expression levels of Bcl-2 and Bcl-xl when ARPE cells were treated with plumbagin at 15 μM for 0, 12, 24 and 48 h. **f** The expression levels of Bax, Cytochrome C, Caspase-3 and Caspase-8 determined by ELISA when ARPE cells were treated with plumbagin at 0,5,15 and 25 μM for 48 h

Since we have known the anti-proliferative effect of PLB on ARPE cells, we next assessed the expression of related proteins responsible for this phenomenon. Many factors are involved in drug-induced apoptosis, and the Bcl-2 family is a critical one. We examined the expression of pro-apoptotic proteins (Bax, Bak) and anti-apoptotic proteins (Bcl-2, Bcl-xl) in ARPE cells by Western blot analysis (Fig. 4a). After treatment with 5, 15, and 25 μM PLB for 24 h, the level of Bax was increased by 1.7-, 2.1-, and 2.4-fold and the level of Bak was increased by 2.5-, 3.1-, and 4.6-fold, compared to control cells, respectively (each *P*<0.001; Fig. 4b). In contrast, the level of Bcl-2 was reduced by 74.8%, 49.9% and 13.6% respectively (each *P*<0.001; Fig. 4c) while the level of Bcl-xl was decreased by 80.9%, 67.8% and 57.8% compared to control cells (*P* = 0.001, 0.000, 0.000; Fig. 4c). Furthermore, the differences between adjacent groups also have statistical significance (*P* < 0.05) and the changes was dependent on the concentration of PLB (Pearson test; *P*<0.001).

The parallel exams showed that PLB regulates the expression of apoptotic proteins in a time-dependent manner as well (Pearson test; *P*<0.001). Briefly, the level of Bax was increased by 1.4-, 2. 1-and 2.7-fold, while Bak was increased by 2.1-, 3.1-, and 5.0-fold after incubation with 15 μM PLB for 12, 24, and 48 h (each *P*<0.001; Fig. 4d). At the same time, the level of Bcl-2 was reduced by 66.2%, 50.0% and 14.2% (each *P*<0.001; Fig. 4e) and Bcl-xl was decreased by 77.3%, 70.5% and 57.6% compared to the control (*P* = 0.026, 0.006, 0.001; Fig. 4e). The differences between adjacent groups had statistical significance except Bcl-xl group (*P* < 0.05).

Moreover, the ELISA outcomes of Bax showed the same tendency as Western Blotting results, the downstream effect factors in endogenous apoptosis process such as Cytochrome C was increased by 1.49-, 1.66-, and 1.8-fold and Caspase-3 was increased by 1.96-, 2.34-, and 2.51-fold, compared to control cells respectively, after treatment with 5, 15, and 25 μM PLB for 24 h. While the level of Cytochrome C was increased by 1.39-, 1. 76-and 1.90-fold, while Caspase-3 was increased by 1.85-, 2.23-, and 2.40-fold after incubation with 15 μM PLB for 12, 24, and 48 h (each *P*<0.001; Pearson test, *P* = 0.001). However, the content of exogenous apoptosis factor Caspase-8 did not show any significant differences. (Fig. 4f).

Taken together, these results demonstrated an up-regulating role in the expression of pro-apoptotic proteins and a down-regulating role in the expression of anti-apoptotic proteins of PLB in ARPE cells.

### PLB suppresses P38MARK and PI3K axis in ARPE cells

**Fig. 5:**
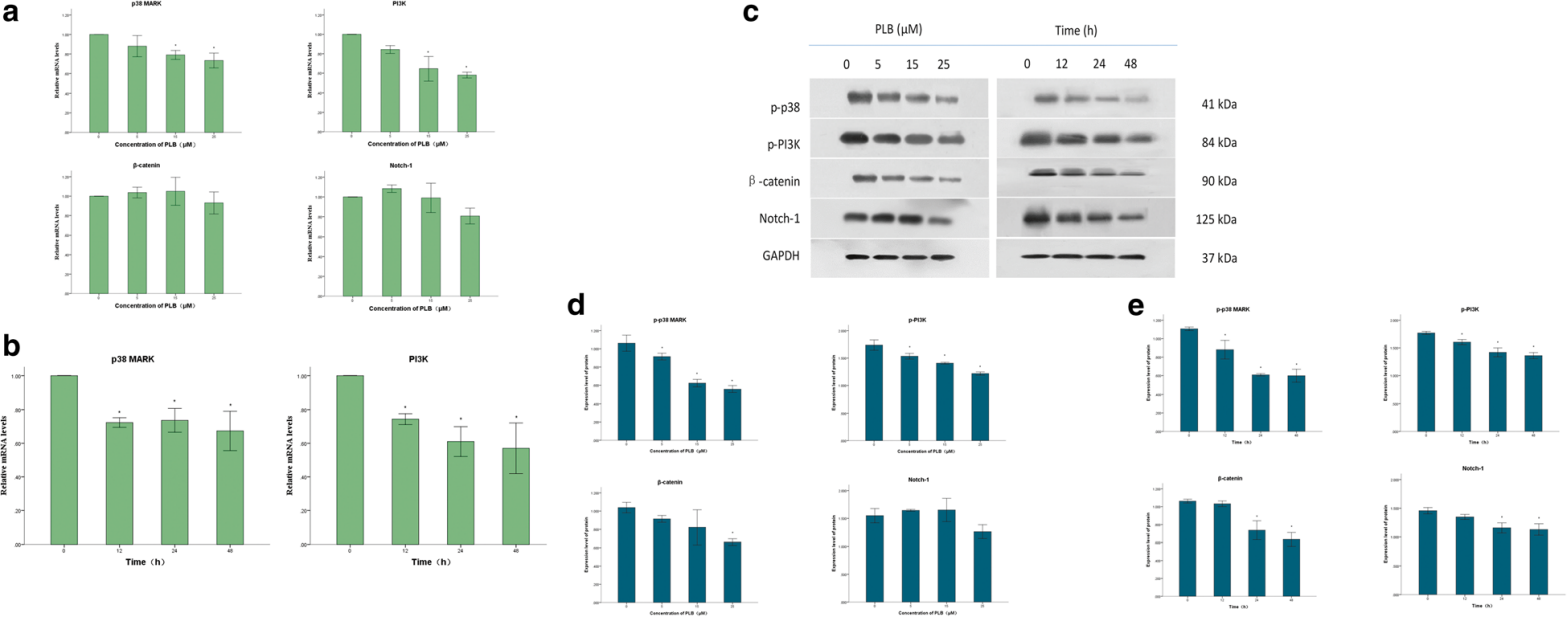
Effect of plumbagin on the different signal pathways in ARPE cells. (*:*P* < 0.05 by one-way ANOVA, *n* = 3). Notes: (**a**) The relative mRNA expression levels of p38 MARK, PI3K, β-catenin and Notch-1 determined by QPCR when ARPE cells were treated with plumbagin at 0,5,15 and 25 μM. β-actin was used as the internal control; (**b**) The relative mRNA expression levels of p38 MARK and PI3K determined by QPCR when ARPE cells were treated with plumbagin at 15 μM for 0, 12, 24 and 48 h. β-actin was used as the internal control; (**c**) The protein levels of p-p38 MARK, p-PI3K, β-catenin and Notch-1 determined by Western blotting assay. GAPDH was used as the internal control; (**d**) The relative protein levels of p-p38 MARK, p-PI3K, β-catenin and Notch-1 when ARPE cells were treated with plumbagin at 0,5,15 and 25 μM; (**e**) The relative protein levels of p-p38 MARK, p-PI3K, β-catenin and Notch-1 when ARPE cells were treated with plumbagin at 15 μM for 0, 12, 24 and 48 h

Upon the observation that PLB can inhibit the proliferation and induce cell apoptosis in ARPE cells, next step we tried to explore the underlying mechanisms. It is well known that p38 MAPK, PI3K-AKT-mTOR, Wnt/β-catenin and Notch signaling pathways play an important role in cell proliferation and work actively during the formation and development of PVR. First, we examined the mRNA relative expression of symbolic tic parts of these signaling pathways: p38 MARK, PI3K, β-catenin and Notch-1 using Real-time Quantitative PCR Detecting System. The results showed PLB prominently inhibited the expression of p38 MARK and PI3K mRNA a concentration-dependently (Pearson test; *P* = 0.001, 0.000, respectively). The production of p38 MARK was decreased by 12.0%, 21.0%, and 26.7% when the cells were incubated with 5, 15 and 25 μM PLB, respectively (*P* = 0.232, 0.026, 0.007; Fig. 5a). Similarly, the PI3K level was decreased by 15.7%, 35.4%, and 41.0% in comparison to the control group, respectively (*P* = 0.083, 0.001, 0.000; Fig. 5a). However, the expression of β-catenin and Notch-1 had no significant change (Fig. 5a). Furthermore, when ARPE cells were treated with 15 μM PLB for 12, 24, and 48 h, there was a 27.7%, 26.4%, and 32.7% reduction in p38 MARK mRNA (*P* = 0.006, 0.008, 0.002) while 25.7%, 39.0%, and 43.0% decrease in PI3K mRNA (*P* = 0.032, 0.003, 0.002; Fig. 5b). In addition, these PLB-induced down-regulation of both pathways were in a time-dependent manner (Pearson test; *P* = 0.008, 0.002, respectively). In a word, the above-mentioned results indicated that PLB could affect p38 MARK and PI3K signaling but do not affect β-catenin or Notch signaling pathways in ARPE cells.

We also observed the relevant proteins as symbolic downstream effector of the signaling pathways using Western blotting, to examine whether PLB had the same effect on direct factors of cell proliferation (Fig. 5c). After incubation with PLB at series of concentration, the content of p-p38MARK was significantly decreased from 1.06 at base level to 0.91, 0.62 and 0.56 dose-dependently (*P* = 0.045, 0.000, 0.000; Pearson test, *P*<0.001). Further, the production of p-PI3K was notably descended from 1.74 at the basal level to 1.53, 1.41, and 1.21 dose-dependently (*P* = 0.010, 0.000, 0.000; Pearson test, *P*<0.001). Nevertheless, there was a slight decrease in β-catenin content, but only the reduction at the highest concentration (25 μM) had a statistical significance (*P* = 0.498, 0.125, 0.000). The expression changes of Notch-1 had no statistically significantly differences, neither (*P* = 0.825, 0.795, 0.125; Fig. 5d). In parallel exams, when ARPE cells were exposed to 15 μM PLB for 12, 24, and 48 h, there was a 21.8%, 45.1%, and 46.8% reduction in the content of p-P38 MARK (*P* = 0.009, 0.000, 0.000); 9.6%, 19.8%, and 23.2% decrease in p-PI3K (*P* = 0.027, 0.000, 0.000); 2.8%, 30.2%, and 39.6% decline in β-catenin (*P* = 0.944, 0.002, 0.000); 7.5%, 20.5%, and 22.6% drop in Notch-1 (*P* = 0.346, 0.005, 0.003; Fig. 5e), respectively. Only the levels of p-p38MARK and p-PI3K proteins had statistical significant differences at each time point, and attenuated in a time-dependent manner (Pearson test; *P*<0.001).

In conclusion, PLB has a potent anti-proliferative effect on ARPE cells by inhibiting p38 MAPK and PI3K/Akt/mTOR signaling pathways, not directly blockage of Wnt/β-catenin and Notch signaling pathways.

## Discussion

Proliferative vitreoretinopathy (PVR) remains an enormous challenge for vitreoretinal doctors. Recent efforts have been directed toward the biochemical inhibition of cellular proliferation and membrane formation [10]. Analyses of epiretinal membranes from patients, as well as animal models of experimental PVR, have regarded RPE cells as the key factor in triggering PVR development. Therefore, we choose them as investigated subjects to predict the effects of biochemical drugs on PVR. ARPE-19 is a human RPE cell line which has similar characters of the RPE cells in vivo and is widely used as an in vitro model for investigation [11, 12]. Hence, ARPE-19 cells were used in our present study to find out the efficacy of plumbagin on RPE cells and PVR.

PLB, a natural compound, has shown potent anti-proliferative effects by regulating various signaling pathways related to cell cycle and apoptosis [3, 6–8, 13–17]. However, whether PLB plays a similar role in RPE cells is unknown as yet. In present study, we demonstrated that PLB promoted cell cycle arrest and apoptosis through PI3K/Akt/mTOR- and p38 MAPK-mediated pathways in ARPE-19 cells, which may benefit the prophylaxis of PVR.

Cell cycle is an important part of cell proliferation including a long growth phase (G1), a DNA replicating phase (S), a short growth phase (G2), and cell division (mitosis, M), and any abnormality in cell cycle may cause apoptosis [18]. We assess the influence of PLB on cell cycle distribution of ARPE-19. Our results from flow cytometry indicate that PLB causes cell cycle arrest at G2/M phase concentration- and time-dependently, consequently prevents cell proliferation and induce cell apoptosis [19].

Apoptosis is a precisely programmed procedure of cell death in order to maintain cellular homeostasis [20]. However, RPE cells in PVR proliferate in an uncontrolled manner and are resistant to apoptosis. We observed that PLB had the capacity to sensitize ARPE cells toward apoptosis. Moreover, the higher of the concentration, the longer of drug duration, there were more numbers of apoptotic cells from early stage to late stage. Treated with 25 μM PLB for 24 h or with 15 μM PLB for 48 h, percentage of apoptotic cells were both over 75%. Herein, PLB showed a remarkable ability to promote apoptosis. These results are consistent with previously published reports suggesting the apoptosis inducing potential of PLB in various cell lines [7, 13, 17].

At a molecular level, the Bcl-2 family proteins regulate the intrinsic apoptosis by changing the permeability of mitochondrial membrane [21]. The pro-apoptotic members Bax and Bak release apoptogenic molecules (such as cyt C), leading to caspase activation, while the anti-apoptotic members, such as Bcl-2 and Bcl-xL, restrain Bax and Bak [22]. In our experiment, PLB possesses an up-regulating ability in the expression of pro-apoptotic proteins and a down-regulating role in the expression of anti-apoptotic proteins of PLB in ARPE cells. The downstream effectors such as Cytochrome C and Caspase-3 changed accordingly. In fact, plenty of previous studies have demonstrated the similar effect of PLB on other cancer cell lines [7, 23]. Thus, it could be inferred that the modulation effect of PLB on Bcl-2 family proteins plays an important role in promoting apoptosis in ARPE cells.

At a gene signal level, PVR is a complex process involving intricate interplay between multiple signaling pathways, such as p38 MAPK, PI3K-AKT-mTOR, Wnt/β-catenin and Notch. In the present study, we have explored that plumbagin suppressed the expression of p38 MAPK and PI3K, which may be the key contributors to ARPE apoptosis.

Mitogen-activated protein kinases (MAPKs) play a momentous role in controlling cell proliferation as signal transducing enzymes, while p38 MARK is one of the key parts [24]. Yamaguchi, K. et al. [25] has reported it functioned in rat cultured RPE cells and its inhibitor could arrest cell cycle progression. In our study, PLB dose- and time-dependently inhibited the expression of p38 MARK mRNA and protein, then reduced the proliferation of ARPE cells. Thus, the restraint of p38 MARK signaling pathway may partly contribute to the affect of PLB in ARPE cells.

In our study, we also observed that PLB predominantly inhibit PI3K-AKT-mTOR axis in the same manner. The PI3K/Akt/mTOR signaling is an important pathway related to cell survival. Normally, PI3K activates serine/threonine kinase Akt, then trigger serine/threonine kinase mTOR, at last inhibit cell death and promote cell survival [26]. It has been stated that thrombin promotes RPE cell proliferation through this PI3K/Akt/mTOR pathway [27]. Also, Liegl R. et al. [28] reported inhibition of mTOR pathway with temsirolimus could consistently suppress proliferation and migration of RPE. Our results indicated that PLB decreased PI3K expression on RNA and protein levels significantly. We therefore consider that PLB act as an inhibitor of PI3K/Akt/mTOR signaling pathway as well in ARPE cells. What’s more, AKT is also a G2-M initiator, which may explain the G2/M arrest of ARPE cells exposed to PLB. Till now, several investigators have revealed that PLB exhibited its apoptosis-inducing effect by inhibition of PI3K/Akt/mTOR way in other cell lines [3, 9, 29], which are consistent with our consequence.

In fact, there are several other signaling pathways in the proliferation of RPE cells, such as Wnt/β-catenin, Notch, Rho-ROCK and so on. Recent studies suggest suppressing the Wnt/beta-catenin signaling pathway may inhibit the propagation of RPE cells [30, 31]. Liu W. et al. [32] also reported the blockage of Notch signaling inhibited the proliferation of RPE cells. On the other hand, previous studies have revealed that PLB was able to restrain Wnt/β-catenin and Notch signaling pathways. Nevertheless, in our study, PLB has not exhibited an obvious blockage on both signaling pathways in ARPE cells. The numerical fluctuations may be due to the cell number changes, or because of crosstalk and interaction between different signal networks, which need more complicated study to explore.

## Conclusions

This study shows that plumbagin (1) induces apoptosis in ARPE cells in vitro; (2) the apoptosis may be associated with G2/M cell cycle arrest; (3) plumbagin inhibits cell proliferation by increasing pro-apoptotic proteins and decreasing anti-apoptotic proteins; (4) plumbagin induces ARPE cell death by inhibition of the p38 MARK and PI3K/AKT/mTOR pathways. These data provide primary evidence for the usage of PLB in PVR. Further in vivo studies are needed to ascertain the chemotherapy effects of PLB and more detailed mechanism should be elucidated in the future.

## Abbreviations

DAPI: 4, 6-diamidino-2-phenolindole
DMSO: Dimethyl sulfoxide
GAPDH: Glyceraldehyde-3-phosphate dehydrogenase
MAPKs: Mitogen-activated protein kinases
MTT: 3-(4, 5 dimethyl-thiazol-2-yl)-2, 5-diphenyltetrazolium bromide
PLB: Plumbagin
PVDF: Polyvinylidene difluoride
PVR: Proliferative vitreoretinopathy
RPE: Retinal pigment epithelial
SDS-PAGE: Sodium dodecyl sulfate polyacrylamide gel electrophoresis minigel
